# Sensory, Physical, and Functional Properties of Part‐Skim, Pasta Filata Mozzarella Made With or Without *Lacticaseibacillus casei* Adjunct Culture

**DOI:** 10.1111/1750-3841.71095

**Published:** 2026-05-08

**Authors:** Katherine Rehberger, Stephanie Clark, Gülhan Ünlü, Helen Joyner, Carolyn F. Ross

**Affiliations:** ^1^ School of Food Science Washington State University Pullman Washington USA; ^2^ American Dairy Products Institute Elmhurst Illinois USA; ^3^ Premier Nutrition Emeryville California USA

**Keywords:** adjunct culture, lactic acid bacteria, *Lactobacillus casei*, meltability, secondary cultures, storage

## Abstract

With increasing mozzarella consumption in the United States, mozzarella innovations are of interest, particularly in functional attributes. One opportunity in mozzarella is the application of *Lacticaseibacillus casei* adjunct culture to increase desirable flavors and modulate functional properties such as melting. The objective of this study was to determine the influence of the adjunct culture, *Lacti. casei*, on the sensory, physical, and functional properties of part‐skim, pasta filata mozzarella cheese. Two treatments of mozzarella were produced: a control with *S. thermophilus*, and a modified treatment with *Streptococcus thermophilus* and *Lacti. casei*. Samples were stored at 3.3°C and assessed at 5, 25, 50, and 75 days in cubed cold and melted preparations by a trained sensory panel (*n* = 10). Meltability and physical properties were assessed through instrumental measures. The inclusion of *Lacti. casei* adjunct modulated melting behavior in baked applications over storage time. Results showed that *Lacti. casei* suppressed mozzarella meltability at Day 25 (compared to the control [*p* ≤ 0.05]). *Lacticaseibacillus casei* did not influence the sensory or texture attributes of cubed cold or melted mozzarella (*p* ≥ 0.05). However, storage time strongly influenced yellow color, butter flavor, bitterness, adhesiveness, and cohesiveness (*p* ≤ 0.05). These findings suggest that *Lacti. casei* may be a beneficial adjunct culture for improving baked mozzarella performance over its shelf life, though it may not be desired for all cheese producers.

## 1 Introduction

Consumption of natural cheese by US consumers has increased by 50.5% over the past 28 years, with the average American consuming 40.5 pounds (18.37 kg) of cheese per year in 2023 (USDA ERS 2024). As part of this 50.5% increase, overall mozzarella consumption has increased by 57.6%, from 7.9 pounds (3.6 kg) to 12.5 pounds (5.7 kg) pounds per year per person (USDA ERS 2024). This makes mozzarella, including all variations such as LMPS, reduced fat, and lactose free, the most popular category of cheese by consumption in the United States. While popular as string cheese, mozzarella is a major component in many baked products, especially pizza. On any given day of the year, 11% of Americans consume pizza (USDA Agricultural Research Service 2024). Mozzarella cheese is considered essential in the pizza industry due to its unique baking properties, including melting, blistering, and browning (Ma et al. 2014). The most common type of pizza or baked applications is low‐moisture part‐skim (LMPS) mozzarella, where lower moisture and lower fat content allow longer storage while still maintaining the important blistering and browning properties that are key in pizza (Ma et al. 2013). Other variations, such as reduced fat, fat free, or lactose free, have been created to fit market needs following consumer trends, though these variations sacrifice ideal mozzarella properties (Natrella et al. 2022a; Sutariya et al. 2022). Current mozzarella innovations modify formulation with novel ingredients (Abdalla et al. 2022) and traditional additive modulations (Grossbier 2023), which impact the sensory, physical, and functional attributes. Adjunct or secondary cultures remain largely unexplored in mozzarella production.

In cheesemaking, a starter culture is added to milk with the main purpose of acidifying the milk. Secondary or adjunct cultures can also be added to modify flavor or other sensory or functional properties of the cheese throughout storage through metabolic processes (Irlinger et al. 2017). A popular adjunct culture is *Lactobacillus helveticus*, known for its ability to metabolize bitter peptides through proteolysis in hard, ripened cheeses such as Cheddar and Dutch cheeses (Garbowska et al. 2021). This reduction in bitterness stems from the unique proteases and peptidases produced by *Lacto. helveticus*, which target peptides known to induce the bitter sensation (Chelladhurai et al. 2023).

While adjunct cultures are often used in the production of ripened cheeses to enhance flavor development and texture through proteolysis, their application in short shelf‐life cheeses such as mozzarella has traditionally been limited. However, growing interest in improving qualities of mozzarella, such as baking applications and flavor enhancement, has renewed attention on the use of adjunct cultures in these systems (Braghieri et al. 2015; Bihola et al. 2024). Flavor plays a large role in consumer acceptance of mozzarella, specifically in the mild, buttery, and creamy flavors associated with it. Buttery flavor in mozzarella has been seen to be attractive, and mild flavor has been seen as a must‐have for mozzarella by consumers (Homwongpanich et al. 2025). One adjunct culture, *Lacticaseibacillus casei*, has been previously identified as contributing to sensory attributes in dairy products with increased diacetyl and acetoin production through citrate uptake and metabolism (Branen and Keenan 1971). *Lacticaseibacillus casei* has also been used as an adjunct culture in yogurt (Dimitrellou et al. 2019), Cheddar cheese (Martinovic et al. 2013), goat milk yogurt (Bezerril et al. 2022), and Italian cheeses (Briggiler‐Marcó et al. 2007). Only one previous study utilized *Lacti. casei* as an adjunct culture in mozzarella, which found that the use of adjunct culture produced a cheese with a denser structure and reduced stretch, with no significant effect on melting behavior. However, the study reported no sensory analysis and limited functional analysis, as it focused on stretch and melt (Merrill et al. 1996).

The objective of the present study was to determine how the inclusion of *Lacti. casei* adjunct culture influences the sensory, physical, and functional properties of part‐skim, pasta filata mozzarella cheese. Of interest was the impact of adjunct culture on cheese aroma, flavor, body, and texture when cold and warm. We hypothesized that:

1) The modified mozzarella would have increased buttery sensory attributes compared to the control mozzarella.
2) When heated, the modified mozzarella would have increased melting compared to the control mozzarella.
3) Modified mozzarella will have reduced TPA hardness, sensory in‐hand firmness, and first‐chew hardness over storage time.

## 2 Methods

### 2.1 Mozzarella Cheese Production and Storage

Triplicate batches of mozzarella cheese were produced over three production days for each treatment (control and modified) at a pilot plant scale. Treatments were randomly assigned to one of the three production days. For each batch, pasteurized whole milk (Knott Dairy Farm, Pullman, WA) and pasteurized nonfat milk (Glenview Farms, West Linn, OR) were mixed, totaling volumes between 88.01 and 104.40 kg per batch, as seen in Table 2. Whole milk was pasteurized at 72.7°C for 15 s, and nonfat milk was high‐temperature short‐time (HTST) pasteurized prior to purchase. Milk was standardized to a goal fat percentage of 3.0% (w/w) using Pearson's square to calculate the needed ratio of nonfat milk to whole milk. Verification of fat percentage was performed using a LactiCheck LC‐03 (Page & Pedersen, Hopkinton, MA), resulting in a mean fat percentage of 2.85 ± 0.127% (w/w).

For each batch of control or modified mozzarella, milk was added to a jacketed X vat and warmed to 35°C, then inoculated with CHOOZIT Swift 522 FRO 375 DCU *S. thermophilus* starter culture (International Flavors and Fragrances, Inc, New York, NY) at a manufacturer‐suggested rate of 9 Danisco Culture Units (DCU) per 100 kg of milk, which converted to a ratio of 7.5 g of culture mix per 100 kg of milk. For the production of the modified mozzarella, milk was also inoculated with the adjunct culture, CHOOZIT Flavobac LC20 FRO 365 DCU *Lacti. casei* (International Flavors and Fragrances, Inc, New York, NY), at a rate of 5 DCU per 100 kg of milk, which converted to a ratio of 5 g of culture mix per 100 kg of milk. Following inoculation, cultures were allowed to ripen for 30 min.

Coagulation ensued with the addition of CHY‐MAX Extra Milk Rennet (Chr. Hansen, Hørsholm, Denmark) at a rate of 15 mL (minimum 600 IMCU/mL) coagulant per 100 kg of milk (diluted 1:40 with cool tap water), followed by gentle stirring (1 min) and setting for 30 min. The curd was then cut with 63.5‐mm wire cheese knives and allowed to heal for 5 min. The temperature of the curd–whey mixture was raised gradually (∼1°C every 5 min) and the mixture was cooked at 43°C until the pH of the whey reached 6.3, at which point a third of the whey was drained off and replaced with tap water. This exchange, known as curd washing, encourages more syneresis of lactose from curd and decreases the galactose concentration, acting as a pH control and further reducing the moisture content for low‐moisture mozzarella (Joshi et al. 2003; Natrella et al. 2022a). The curd was cooked further at 40°C until the curd pH reached pH 5.3. Whey was drained and the mass was trenched, cut into loaves, and flipped twice. Curd loaves were cut into strips (5‐cm wide, 36‐cm long), then stretched by hand at an average pH of 5.34 ± 0.06 in 55°C–65°C water. Strips were stretched to shoulder‐width apart and folded four times, then formed into balls. Cheesemaking time was consistent across vats, between 2.5 and 3 h, regardless of treatment. Balls were placed into cool water for at least 10 min, then into concentrated brine.

The 25% (w/v) NaCl and 0.15% (w/v) CaCl_2_ brine was prepared 24 h prior to the first production day using MORTON Top Flake Extra Course Salt (Morton Salt, Chicago, IL) and 32% (w/v) CaCl_2_ solution (Nelson Jameson, Marshfield, WI). The pH of the brine was adjusted to 5.3 using 50% (w/v) citric acid. Mozzarella balls were brined for 12 h at 3.3°C. Each batch produced between 24 and 26 balls of cheese, with weights ranging from 481.8 to 658.7 g per ball (mean of 555.8 ± 56.9 g).

After removal from brine, balls were dried and vacuum‐sealed in plastic bags in pairs using an industrial vacuum sealer (VacMaster Commercial Double Chamber Vacuum Sealer, Overland Park, KS). Cheese balls from each production day were stored at 3.3°C. At each sampling date, a set of four balls of cheese from each of the three production days of control and modified mozzarella were randomly selected for assessment, with two balls used for analytical measures and two balls for sensory testing.

### 2.2 Composition Measurements

At each time point, one ball of mozzarella from the control and one from the modified treatment made on each of the three production days (*n* = 3 balls from the control treatment and *n* = 3 balls from the modified treatment) were cubed using a smooth‐edged knife for sensory and physical tests or finely grated using a consumer kitchen grater or home food processor (Cuisinart, Stamford, CT). All measurements were taken as replicates from each replicate ball, resulting in a total of 12 measurements at each time point (Days 5, 25, 50, and 75).

For each batch, one whole ball of mozzarella was shredded for use in the FoodScan analysis, the Babcock method, and the oven‐drying method. The moisture content of the mozzarella samples was assessed using an oven‐drying method, with a BLUE M C‐4850‐Q forced air oven (Thermal Product Solutions, New Columbia, PA) (Marshall 1992). Moisture content was calculated as the difference in weight between the sample predrying and postdrying, expressed in grams and converted to a percentage. Fat was determined using the Babcock method (Marth 1978). To determine salt and protein content, samples were analyzed by filling half a petri dish to the level and then placing it into the FOSS FoodScan 2 Dairy system (FOSS Analytical A/S, Hillerød, Denmark). Samples were analyzed in duplicate and referenced using the FOSS mozzarella database (Rathod et al. 2025; Małkowska‐Kowalczyk et al. 2024).

### 2.3 Adjunct Culture Enumeration

Microbiological enumeration was conducted at time points of 5, 15, 35, and 50 days of storage in order to ensure consistent growth and recovery of lactic acid bacteria populations. One ball of mozzarella from the control and one from the modified treatment made on each of the three production days (*n* = 3 balls from the control treatment and *n* = 3 balls from the modified treatment; prepared as replicates) were prepared for microbial enumeration. Grated cheese was added to sterile 2% (w/w) sodium citrate buffer (pH 7.0) and stomached for 4 min at 300 rpm using a Stomacher 400 Circulator (Seward Laboratory Systems Inc., Bohemia, NY) (Oberg et al. 2011). Dilutions were prepared in sterile 2% (w/w) sodium citrate buffer (pH 7.0) to a final dilution of either 10^−8^ or 10^−9^. Each dilution was plated in duplicate on de Man, Rogosa, and Sharpe (MRS) (Hardy Diagnostics, Santa Maria, CA) agar and MRS‐Vancomycin (MRS‐V) agar (Oberg et al. 2011). Plates were anaerobically incubated for 72 h at 37°C. Resulting colonies were counted and reported as log colony‐forming units (CFU) per milliliter. Plates with 30–300 colonies were used. Further basic identification of select colonies was done through Gram staining. By Day 50, the microbial counts reached a stable phase; therefore, additional sampling was not conducted.

### 2.4 Texture Profile Analysis (TPA)

At each time point, samples (*n* = 3 balls from the control treatment and *n* = 3 balls from the modified treatment; prepared as triplicates) were prepared for TPA using an AMETEK Brookfield CTX Texture Analyzer (AMETEK Brookfield, Middleborough, MA). To prepare for analysis, cheese was cut using a smooth‐edge knife into rectangular prisms, 13 mm (*l*) × 13 mm (*h*) × 17 mm (*w*). Due to the pasta filata structure of the cheese, the prisms were oriented with stretched strands running perpendicular to the compression, allowing for consistency across samples (Villanueva‐Carvajal et al. 2012).

Samples were compressed to 62% of the total height as measured by distance traveled using a 50‐kg load cell, TA25/1000 probe, and the FA‐ABT fixture in a two‐bite compression test (Moynihan et al. 2014). The probe pretest speed was set to 1.00 mm/s, test speed to 0.80 mm/s, and posttest speed to 1.00 mm/s. The test was performed on cheese stored at 3°C, and no moisture was exuded during compression. Measurements were collected from the two‐bite compression test. Physical TPA measurements of hardness (g), springiness (unitless), gumminess (g), chewiness (mJ), adhesiveness (mJ), and cohesiveness (unitless) were selected from the two‐bite compression test, as these attributes are commonly reported in mozzarella. These measurements are distinct quantifiable mechanical outputs but can be related to sensory attributes such as firmness, in‐hand springiness, and chewiness.

### 2.5 Melting

At each time point, one ball of mozzarella from the control and one from the modified treatment made on each of the three production days were used to assess the melting properties of the cheese using a modified Schreiber test (*n* = 3 balls from the control treatment and *n* = 3 balls from the modified treatment; prepared as triplicates) (Muthukumarappan et al. 1999). Samples were heated in a Precision Scientific Laboratory Oven for 5 min at 90°C (Thermo Fisher Scientific, Waltham, MA). Cheese samples were sliced into 2.1‐cm‐thick slabs, then cut into 3.5‐cm‐diameter discs using a stainless‐steel ring. Samples were then placed on an aluminum pan with a ruler, and a “pre‐melting” image was captured from 60 cm above using an iPhone 15 (Apple Inc., Cupertino, CA) at 1.0 zoom. “After‐melting” images were captured after 5 min of resting at 22°C, following the same procedure as the “pre‐melting” imagery. From the images, the final melted area was measured using ImageJ (NIH, Bethesda, MD, and Laboratory for Optical and Computation Instrumentation, Madison, WI). The scale for each image was set by marking a 30‐cm line using the ruler and converting the pixel count to centimeters. The spread of area for each sample was measured using the polygon tool by tracing the edge of the melted cheese, resulting in the final melted area. Results were expressed as final melted area (cm^2^).

### 2.6 Trained Sensory Evaluation Panel

At each time point, two balls of mozzarella from the control and modified treatment made on each of the three production days were used by a trained panel to assess the sensory attributes of the cubed cold and shredded melted mozzarella samples (*n* = 6 balls from the control treatment and *n* = 6 balls from the modified treatment). Volunteers (*n* = 10, aged 26–57 years, mean age = 40.2) were recruited through the Washington State University Sensory Science Center listserv and social media posts. No previous experience in trained panels was required. The panel consisted of eight female, one male, and one gender nonconforming panelists. Panelists were initially trained for 20 h before the first evaluation (5 weeks of 1.5‐h sessions). Prior to each subsequent time point (Days 5, 25, 50, and 75), panelists participated in a 1‐h refresher session. Panelists were not informed of the nature of the study to reduce bias. The study was approved for participation by human subjects by the Washington State University Institutional Review Board for human subject participation under IRB# 20397‐001.

During training sessions, panelists were trained on 18 attributes describing the appearance, aroma, basic taste, flavor, and texture of cubed cold and shredded melted mozzarella. The mozzarella flavor and aroma lexicon was adapted from Pagliarini et al. (1997) and Braghieri et al. (2018), while the texture attributes were adapted from Chen et al. (2008) and Brown et al. (2003). Panelists were instructed to rate these attributes on a semistructured 15‐cm line scale, with anchors at 1.5 cm (low) and 13.5 cm (high). Reference standards (Table 1 ) were presented for each attribute during training and scaled on the 15‐cm line scale through consensus. Panelists performed practice evaluations on cubed cold and shredded melted commercial cheese samples, which were used to generate performance feedback using the *Compusense Cloud* (Compusense Inc., Guelph, ON, Canada) postsession feedback and SenPaq (Qi Statistics Ltd., Berkshire, UK). Refresher sessions were held the day before final evaluations, including practice evaluations on commercial cheeses, panelist discussions, and panelist feedback.

**TABLE 1:** Standards used in training panelists (*n* = 10) for sensory evaluation of mozzarella cheese samples for appearance, aroma, flavor, basic taste, and texture attributes.

Attribute	Definition and assessment technique	Standard; intensity along a 15‐cm line scale
Appearance		
White color	Degree of white to cream color in a given sample.	*Valspar Paint Chips* Fossil White; 4.0 Dairy Belle; 11.0
Yellow color	Degree of yellow color to orange in a given sample.	*Cloverdale Paint/Rodda Paint Chips* 0929; 3.0 0930; 7.5 0931; 12.0
Surface uniformity^a^	Product surface free of holes, cracks, and granules	Galbani Fresh Mozzarella, 3.0 Great Value Monterey Jack, 12.0
Basic tastes		
Salt^a^	Basic taste sensation elicited by salt	0.8% (w/v) salt (NaCl) solution; 10.0
Acid^a^	Basic taste sensation elicited by acid	0.14% (w/v) citric acid solution; 10.0
Bitter^a^	Basic taste sensation elicited by caffeine	0.18% (w/v) caffeine solution; 10.0
Aroma/flavor		
Milk aroma/flavor^a^	Characteristic aroma/flavor arising from whole milk at room temperature	80% (v/v) Lucerne whole milk 20% (v/v) water; 10.0
Butter aroma/flavor^a^	Characteristic aroma/flavor arising from butter at room temperature	34% (w/w) mascarpone, 66% (w/w) unsalted Tillamook butter; 10.0
Yogurt aroma/flavor^a^	Characteristic aroma/flavor of plain whole milk yogurt at room temperature	80% (w/w) Dannon whole‐milk yogurt, plain, 20% (w/w) Lucerne Whole Milk; 10.0
In‐hand texture		
Hand firmness^b^	Force required to compress the cheese between finger and thumb. Place the cheese cube between thumb and forefinger. Compress cheese cube; do not fracture.	Great Value sharp Cheddar, 2‐cm cube served at 4°C; 10.0
Hand springiness^b^	The total amount of recovery of the sample. Press the sample between thumb and two first fingers until it is depressed 30%.	Great Value sharp Cheddar, 2‐cm cube served at 4°C; 7.0 Great Value muenster, 2‐cm cube served at 4°C; 13.0
In‐mouth texture		
First chew: Hardness^b^	Force required to bite entirely through the sample with molars.	Great Value sharp Cheddar, 2‐cm cube served at 4°C; 6.0
Chew down: Adhesiveness of mass^b^	Degree to which mass sticks to the roof of the mouth or teeth. Chew cheese sample between molars 12–15 times and assess adhesiveness.	Great Value sharp Cheddar, 2‐cm cube served at 4°C; 11.0
Chew down: Cohesiveness of mass^b^	Degree to which sample holds together in a mass. Put cheese sample between molars and chew 15 times. Gather to the middle of mouth and evaluate the cohesiveness of mass.	Great Value sharp Cheddar, 2‐cm cube served at 4°C; 11.0
Chew down: chewiness^c^	The length of time required to masticate the sample to a state pending swallowing. The longer the time required, the chewier the sample is.	Great Value muenster, 2‐cm cube served at 4°C; 6.0

^a^ Braghieri et al. (2018).

^b^ Brown et al. (2003).

^c^ Moynihan et al. (2014).

**TABLE 2:** Volumes of whole milk, nonfat milk, and total milk used in the production of each batch of Mozzarella cheese.

Treatment	Replication	Whole milk (kg)	Nonfat milk (kg)	Total mixed milk (kg)
Control mozzarella with no added adjunct culture	1	62.77	26.72	89.49
Control mozzarella with no added adjunct culture	2	66.10	23.21	89.31
Control mozzarella with no added adjunct culture	3	75.96	28.44	104.4
Modified mozzarella with added adjunct culture	1	61.28	28.69	89.97
Modified mozzarella with added adjunct culture	2	64.72	23.29	88.01
Modified mozzarella with added adjunct culture	3	74.99	28.08	103.07

Final evaluations were performed at Days 5, 25, 50, and 75 of storage to reflect the general 3‐month storage of mozzarella through *Compusense Cloud* (Compusense Inc., Guelph, ON, Canada), and all samples received randomized three‐digit blinding codes.

Cubed cold cheese samples were cut into 2‐cm cubes, and five cubes were placed into lidded 4‐oz soufflé cups. Cubed cold samples were served directly out of refrigeration (4°C), similar to serving string cheese. Shredded, melted cheese samples were prepared by first shredding using a home food processor (Cuisinart, Stamford, CT). A sample of this cheese (20 g) was melted in a ceramic ramekin for 15 s at 1250 W in a microwave (Panasonic, Kadoma, Osaka, Japan), transferred into the 4‐oz soufflé cup, lidded, and then immediately served to panelists. Panelists were provided with green grapes, unsalted crackers, and water as palate cleansers, with forced 60‐s breaks between samples and a 15‐min break after six samples to prevent fatigue. Panelists received unique randomized serving orders. Results were expressed as intensity ratings along the 15‐cm line scale.

### 2.7 Statistical Analyses

All data were analyzed using XLSTAT Basic 2024.2.2 (Addinsoft, Paris, France).

Milk fat percentage (w/w) was analyzed using a two‐way analysis of variance (ANOVA) with treatment and production day as main effects. Proximate analysis data (including fat, protein, moisture, salt), melting data, and TPA were analyzed using a three‐way ANOVA, with treatment, storage time, and batch as main effects. For each of these measurements, the interaction between treatment and storage time was determined. An additional two‐way ANOVA with replicate and storage time was run. Mean comparison was completed using Tukey's HSD, with significance defined as *p* ≤ 0.05.

Trained sensory evaluation data of cubed cold and shredded melted mozzarella were analyzed through two models. First, a two‐way ANOVA with storage time and production day batch as the main effects was run for each panelist to identify the influence of replicate within each panelist on assessments. Production day batch represented a replicate, as three evaluations of the same treatment were performed per panelist. Next, a three‐way ANOVA with treatment, storage time, and panelist included as main effects was run, including the interaction between treatment and storage time. Mean separation was accomplished using Tukey's HSD, with significance defined as *p* ≤ 0.05. Principal component analysis (PCA) was used to visualize the relationship of sensory attributes as the interaction of treatment with storage time. Partial least squares (PLS) regression was used to visualize the correlation between TPA physical properties and sensory texture evaluations.

## 3 Results and Discussion

### 3.1 Compositional Analysis

Milk fat percentage (w/w) was not found to be significantly different (*p* ≥ 0.05) through a two‐way ANOVA between treatment and production day (Table S1). The cheese samples were ≤45% fat in dry matter (w/w%), and the moisture content of both treatments was between 53% and 55% (w/w). The control mozzarella had an average moisture content of 54.62% (w/w), significantly (*p* ≤ 0.05) different from the modified mozzarella's average moisture content of 53.45% (w/w). Similarly, the FDM in the control mozzarella was 43.40% (w/w) compared to the significantly (*p* ≤ 0.05) lower 42.57% in the modified mozzarella. The protein content was lower (*p* ≤ 0.05) in the modified cheese at 18.80% compared to the control mozzarella at 19.63%, though the difference between these values was low (≤1%). Under the Code of Federal Regulations for mozzarella (US Government Publishing Office 1993a), the mozzarella produced in the present study was considered part‐skim mozzarella due to its higher moisture content than LMPS mozzarella (US Government Publishing Office 1993b). This was most likely due to the rapid decrease in pH during cheese production, decreasing time for syneresis, thus resulting in a higher moisture cheese (Hutkins 2006; Gunasekaran and Ak 2003).

Storage time influenced the composition of the mozzarella cheese. After 50 days of storage, the average final pH of all treatments and replicate batches was 4.96 ± 0.47. Over storage time, protein concentration differed between the treatments significantly (*p* ≤ 0.05), as detected by the FOSS system. The reported salt levels may be higher than expected in mozzarella due to the extended brining process, as salt content has been found to increase with extended brining in mozzarella cheese over 24 h (Luo et al. 2013). Salt concentration (w/w%) increased over time in the control cheese, from 2.27% (w/w) at Day 5 to 3.15% (w/w) at Day 75 (*p* ≤ 0.05). The salt concentration in the modified mozzarella cheese also increased over time, from 2.39% (Day 5) to 3.46% (Day 25) and 3.06% (Day 75). This behavior has not previously been observed in mozzarella, though salt does impact proteolysis over time and has been found to migrate throughout the mozzarella blocks previously (Luo et al. 2013; Cervantes et al. 1983; Kindstedt et al. 1992).

### 3.2 Microbiological Profile

**FIGURE 1:**
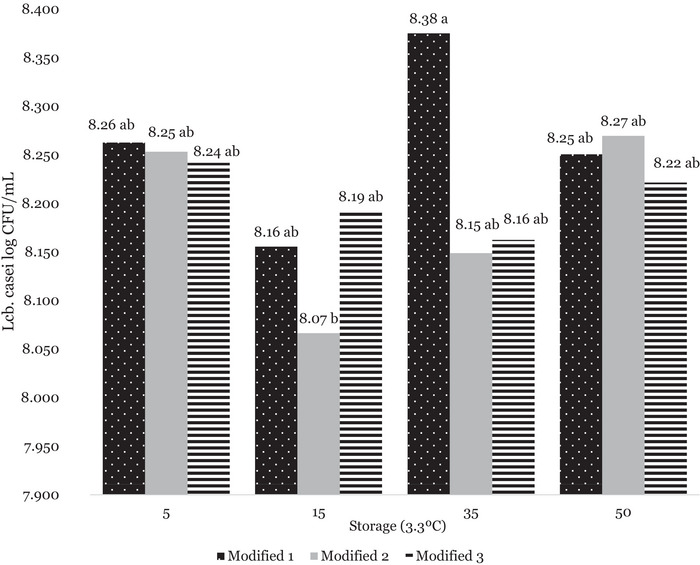
Log CFU/mL of *Lacti. casei* colonies as isolated by MRS‐Vancomycin media over storage (3.3°C) of 50 days, as calculated by colony counts from serial dilution. Data were analyzed using Tukey's HSD. Different letters represent a significant difference among storage times (*p* ≤ 0.05).

Adjunct culture was enumerated in the control and modified mozzarella cheeses at Days 5, 15, 35, and 50 through the MRS‐V (Figure 1). MRS‐V agar follows previously published methods demonstrating successful recovery of *Lacti. casei* from Cheddar cheese (Oberg et al. 2011). No adjunct culture was found in the control samples at any time point on MRS‐V media, demonstrating good separation and sanitation practices. Through Gram staining, the colonies were identified to be Gram‐positive bacilli (rods), consistent with *Lacti. casei* (Huang et al. 2018). The presence of colonies on the MRS‐V media indicates that the *Lacti. casei* culture survived the short‐time, high‐temperature stretching process, which took place in water around 65°C, a higher temperature than the optimal range of 30°C–40°C of *Lacti. casei* as a mesophilic culture (Batt 2014). This high temperature of 65°C is near lethal for *Lacti. casei*, though the exposure time of the curd was below 5 min, and immediate cooling may have mitigated some cell death (Bommasamudram et al. 2022). In the modified mozzarella cheese, significant differences in log CFU/mL were found across storage times, though there was no significant difference between Day 5 and Day 50 of storage. There were no significant differences between different production day batches. The presence of this culture still being active in the mozzarella may support the hypothesis that proteolysis continued over storage, as proteolysis is a key metabolic activity (Tunick et al. 1993).

**FIGURE 2:**
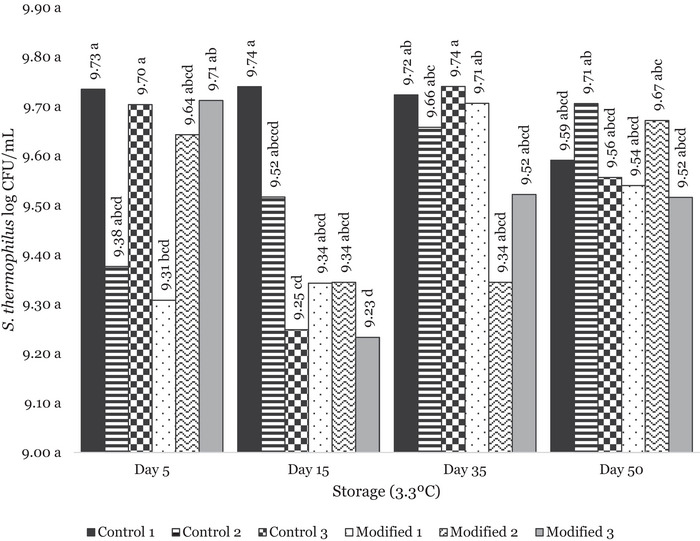
Log CFU/mL of *S. thermophilus* colonies as isolated by MRS media over storage (3.3°C) of 50 days, as calculated by colony counts from serial dilution. Data were analyzed using Tukey's HSD. Different letters represent a significant difference among storage times (*p* ≤ 0.05).

Additionally, the presence of colonies most likely to be the starter culture *S. thermophilus* on the MRS media indicated that these microorganisms survived in all control and modified mozzarella batches (Figure 2). While some variation was observed among batches over storage time, no significant differences were noted between Day 5 and Day 50 of storage (*p* ≥ 0.05). These colonies were subjected to Gram staining, with consistent Gram‐positive cocci arranged in chains being observed. While this is not a full identification of the organism, this Gram stain was consistent with *S. thermophilus* (Roux et al. 2022). As thermophilic lactic acid bacteria, *S. thermophilus* has an optimal range between 35°C and 42°C, higher than the mesophilic cultures (Radke‐Mitchell and Sandine 1986). Additionally, *S. thermophilus* has been found to survive high temperature (between 42°C and 52°C) for longer times, such as 15–30 min, though this induces a shock response (Auffray et al. 1995). Again, while the cheese was exposed to water (∼65°C) during the short stretching process, the detection of the colonies indicates survival. Although older reports suggest limited salt tolerance in *S. thermophilus*, recent cheesemaking studies show that its viability is unaffected even under high‐salt brine conditions. This aligns with our findings, indicating that the elevated S/M levels measured in our mozzarella did not impact the survival of *S. thermophilus* (Wilkinson and LaPointe 2020).

### 3.3 Cold, Cubed Mozzarella Analyses

#### 3.3.1 Trained Panel Sensory Analysis

Mozzarella cheese treatments were profiled by trained sensory evaluation panelists over 75 days of storage at 3.3°C. Samples were profiled both as cubed cheese (served cold) and as shredded, melted cheese (served hot).

Through the evaluation of 18 sensory attributes across appearance, aroma, basic taste, flavor, and texture, no significant differences were found due to the main effect of cheese treatment, that is, between the control and the modified treatment of cold, cubed mozzarella (Table S2). Panelists did have a significant effect, a common result with trained panelists. Although training does mitigate variation between panelists through teaching and consensus, some variation remained in the individual perception of specific attributes. However, storage time significantly influenced the intensity of 10 sensory attributes, including butter aroma, white color, yellow color, surface uniformity, bitter taste, butter flavor, yogurt flavor, hand firmness, chewdown adhesiveness, and chewdown cohesiveness (Table 3). The white color intensity values significantly decreased between the first evaluation day (Day 5) and Days 25, 50, and 75, while the yellow color increased in samples assessed on Day 5 compared to those assessed on Days 50 and 75 (*p* ≤ 0.05). Surface uniformity increased between Day 5 and Day 25. Bitterness in the mozzarella cheese increased in later storage, with a significant increase between Day 25 and Day 50. Butter flavor and yogurt flavor increased earlier, with differences between Day 5 and Day 25 (*p* ≤ 0.05). Additionally, butter aroma increased later, between Days 25 and 50 (*p* ≤ 0.05).

**TABLE 3:** Mean values of significant sensory attributes (identified using three‐way ANOVA) of cubed mozzarella cheese stored for 5, 25, 50, and 75 days at 3.3°C. Mozzarella samples were evaluated by a trained sensory evaluation panel (*n* = 10), with attributes assessed along a 15‐cm line scale for attribute intensity. Data were collapsed across treatment, replicate batches, and panelists, and analyzed using Tukey's HSD. Different letters within a column represent a significant difference among cheese storage times for a given parameter (*p* ≤ 0.05).

	Aroma	Appearance			Taste/flavor			Texture		
Storage day (3.3°C)	Butter aroma	White color	Yellow color	Surface uniformity	Bitter	Butter flavor	Yogurt flavor	Hand firmness	Chewdown adhesive	Chewdown cohesive
5	6.78 ± 1.62 a	9.58 ± 1.13 a	6.47 ± 1.56 a	8.60 ± 2.05 a	4.31 ± 1.47 a	6.11 ± 2.02 a	7.57 ± 1.42 a	6.12 ± 1.56 a	7.55 ± 1.76 a	7.23 ± 1.84 a
25	7.03 ± 1.00 ab	8.90 ± 1.05 b	6.98 ± 1.24 ab	9.39 ± 2.07 b	4.48 ± 1.48 a	6.96 ± 1.22 b	8.02 ± 1.03 b	7.54 ± 1.72 b	8.84 ± 1.86 b	9.19 ± 1.57 b
50	7.23 ± 0.86 b	8.45 ± 0.98 c	7.26 ± 1.28 b	8.60 ± 1.66 a	5.42 ± 1.38 b	7.24 ± 0.99 b	7.81 ± 1.02 ab	6.63 ± 1.66 a	9.05 ± 1.77 b	8.58 ± 2.16 b
75	6.92 ± 1.25 ab	8.63 ± 0.85 bc	7.40 ± 0.75 b	8.85 ± 1.74 ab	5.37 ± 1.33 b	6.88 ± 1.33 b	7.88 ± 1.06 ab	6.71 ± 2.09 a	8.84 ± 1.83 b	8.67 ± 2.04 b

Regarding cheese texture, the firmness as assessed by hand increased during the early storage time (up to Day 25), followed by a decrease at Day 50. The in‐mouth texture attribute of adhesiveness decreased during early storage times, between Day 5 and Day 25 of storage (*p* ≤ 0.05), whereas cohesiveness increased between Day 5 and Day 25 of storage (*p* ≤ 0.05).

**FIGURE 3:**
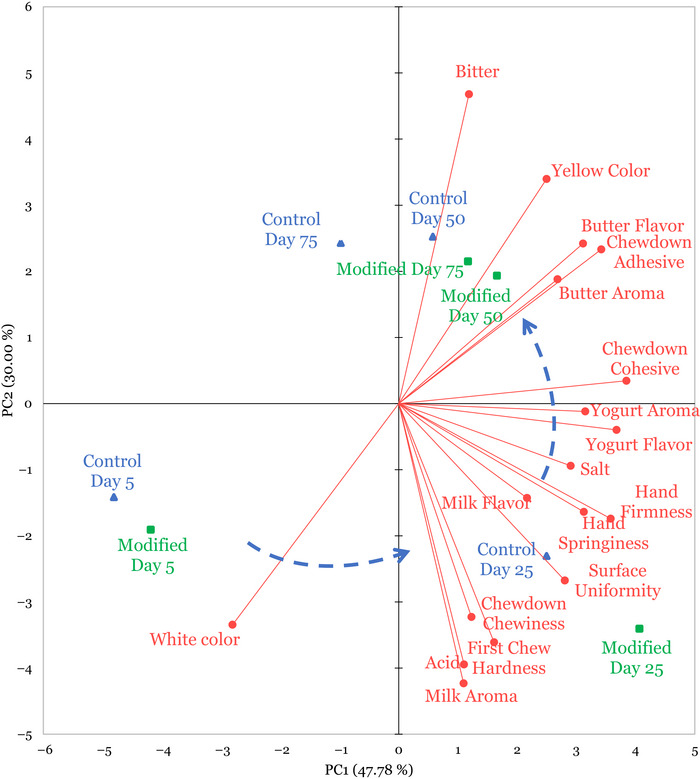
Principal component analysis of cubed mozzarella cheese stored for 5, 25, 50, and 75 days at 3.3°C, as assessed by a trained sensory analysis panel (*n* = 10). Sensory attributes (red vectors) were assessed along a 15‐cm line scale, and results were collapsed by replicate batches. The control mozzarella samples without added adjunct culture are presented in blue, and the modified mozzarella samples with added adjunct culture are presented in green.

To visualize the relationships among the sensory attributes of the control and modified cubed mozzarella treatments, a PCA plot was generated (Figure 3). This PCA accounted 77.78% of the variation among the data, with PC 1 accounting for 47.78% and PC2 accounting for 30.0% of the variation. PC1 was described by yogurt aroma, yogurt flavor, and chewdown cohesive attributes, while PC2 was defined by the contrast between bitterness and acidity, and milk aroma.

Changes in the sensory profile of the cheese during storage may be pictured through movement across the PCA plot. Both mozzarella treatments at Day 5 of storage were in the lower left quadrant, being defined by a high intensity of white color and lower intensities of all other attributes. As storage progressed to Day 25, both treatments moved to the lower right quadrant as they became defined by increased intensities of surface uniformity, milk aroma/flavor, acidity, and texture attributes such as springiness, chewiness, and hardness. These treatments at Day 25 of storage were identified to be lower in bitterness, yellow color, and butter aroma/flavor. At Day 50 and Day 75, the evaluations of mozzarella were similar to each other, as both mozzarella treatments were increasingly defined by the increase in yellow color intensity, bitterness, and cohesiveness, with lower intensities of attributes that defined the earlier storage points.

The sensory properties of the cold mozzarella cheese showed changes with storage time. These differences were expected for part‐skim mozzarella, as previous studies indicated similar sensory changes throughout a 3‐month (∼90 days) period for mozzarella cheese at 4°C (Liu et al. 2024; To et al. 2022). Mozzarella is not a ripened cheese and can be sold at any point during its typical 3‐month storage.

White color has been identified as a positive indicator of attractive and desirable block mozzarella, whereas a pale‐yellow color has been found to be disfavored and disliked (Homwongpanich et al. 2025). Additionally, the intensity of yellow color has been shown to increase in cheese throughout storage, and as a result, the intensity of white color decreases. This may be due to the oxidation of the fat present in the cheese, decreasing the white color and increasing the yellow color in early storage (Alinovi et al. 2020).

Buttery aroma and flavor increased for both treatments of mozzarella over storage time, with the peak buttery notes found at Day 50 of storage, an expected result as diacetyl formation usually takes a few weeks to occur, as previously seen in Cheddar cheese (Martinovic et al. 2013). This increase in buttery aroma and flavor in both treatments of cheese indicates the citrate metabolism process occurring by some *Lacti. casei* cultures. This metabolic process occurs when citrate is taken up by the culture from the cheese matrix and broken down into oxaloacetate and acetate. The oxaloacetate is further broken down into pyruvate and CO_2_, leading to gas production and the further conversion of pyruvate into acetoin, diacetyl, and 2,3‐butanediol (Hugenholtz 1993).

Bitterness increased in both treatments of cheese, though the intensities remained relatively low. This increase in bitterness was expected, as residual proteases from the rennet and active primary cultures allow proteolysis to break down larger proteins into smaller peptides often associated with bitterness (Kindstedt 1993; Tunick et al. 1993). Proteolysis is not only responsible for the increase in bitterness but also for changes in texture. As storage time progressed, both treatments of mozzarella experienced increases in adhesiveness and decreases in hardness (Rathod et al. 2025). Due to the presence of increased bitterness, decreased hardness, and increased adhesiveness, proteolysis of the cheese structure by cultures is assumed. However, it is important to note that previous studies show residual rennet was not completely deactivated during the cheesemaking process and remained active throughout 4°C storage for several weeks, potentially also playing a role in the control and modified mozzarellas (Tunick et al. 1991).

#### 3.3.2 TPA

No significant differences were detected between the two treatments of mozzarella for physical properties as determined by the TPA two‐cycle compression test at any time point (*p* ≥ 0.05) (Table S3). However, storage time exerted an effect on the physical properties of hardness, springiness, gumminess, chewiness, adhesiveness, and cohesiveness (*p* ≤ 0.05; Table 4). These overall changes are all indicative of proteolysis of the casein structure in mozzarella, though the addition of *Lacti. casei* adjunct culture did not seem to accelerate this degradation.

**TABLE 4:** Mean values of texture profile analysis attributes of mozzarella cheese with and without adjunct culture treatment over different storage times (5, 25, 50, and 75 days at 3.3°C). Data were collapsed across treatment and replicate batches, and analyzed using Tukey's HSD. Different letters within a column represent a significant difference among mozzarella cheese samples by storage day for a given parameter (*p* ≤ 0.05).

Storage day (3.3°C)	Hardness on Cycle 1 (g)	Hardness on Cycle 2 (g)	Springiness	Gumminess (g)	Chewiness (mJ)	Adhesiveness (mJ)	Cohesiveness
5	3660 ± 1420 a	2500 ± 860 a	6.03 ± 0.89 a	1440 ± 480 a	83.2 ± 32.1 a	1.89 ± 1.30 a	0.39 ± 0.06 a
25	2440 ± 770 b	1740 ± 560 b	5.04 ± 0.85 b	860 ± 300 b	43.9 ± 20.8 b	2.53 ± 1.04 ab	0.36 ± 0.06 a
50	2580 ± 830 b	1740 ± 560 b	4.66 ± 0.63 bc	790 ± 310 bc	37.3 ± 18.1 bc	2.11 ± 1.05 ab	0.30 ± 0.05 b
75	2140 ± 670 b	1560 ± 465 b	4.19 ± 0.72 c	600 ± 180 c	25.4 ± 9.03 c	3.04 ± 1.13 b	0.29 ± 0.07 b

Specifically, hardness (g) on Cycle 1, the first compression, and Cycle 2, the second compression, decreased significantly between Day 5 and Day 25 of storage, followed by continued decreases with longer storage, though not significantly different beyond Day 25. The change in hardness is characteristic of continued proteolysis by both the starter and adjunct cultures as the casein structure becomes broken down into smaller components (Tunick et al. 1991). The reduction of hardness over storage time is well documented in mozzarella, and the higher moisture content may have accelerated this reduction, as higher moisture mozzarellas have been found to have reduced hardness compared to LMPS mozzarella. The increase in hardness of LMPS mozzarella means a firmer cheese that has less tendency to stick and shreds more evenly, compared to higher moisture cheeses that tend to become soft‐bodied and less efficient in shredding. Hardness is a key attribute in the shreddability of mozzarella, and the degradation of hardness over storage reduces the commercial viability of high‐moisture mozzarella (Banville et al. 2014; Rathod et al. 2025).

Springiness decreased significantly over storage, with large decreases between Day 5 and Day 25 and between Day 25 and Day 75. The loss of springiness over storage also reflects continued proteolysis as the structure is unable to recover to the same degree when compressed. This is an expected behavior of all types of mozzarella cheese over storage (Rathod et al. 2025; Kindstedt et al. 1995). In a similar fashion, gumminess and chewiness both decreased over storage with two large decreases between Day 5 and Day 25 and between Day 25 and Day 75 (*p* ≤ 0.05). Although gumminess is used to describe semisolid food products, in some cases, mozzarella behaves as a semisolid at room temperature (Muliawan and Hatzikiriakos 2007). However, mozzarella can also behave as a solid; therefore, chewiness is often reported. These reductions in gumminess and chewiness match the reduction in hardness and springiness. While it is partly due to calculation methods, these reductions can also be indicative of the continued proteolysis activity (Małkowska‐Kowalczyk et al. 2024). Cohesiveness displayed the same pattern as adhesiveness over storage, decreasing between Day 25 and Day 50 of storage, again partly due to how this parameter is calculated. Although the produced cheese decreased in cohesiveness over storage, most likely due to proteolysis, the cohesiveness values remained high enough not to interfere with the shredding process compared to published cohesiveness values (Banville et al. 2014). Unlike the other physical properties, adhesiveness increased over storage time between Day 5 and Day 75 of storage, which has been observed in mozzarella cheese previously. Increased adhesiveness in mozzarella is undesirable as adhesiveness leads to exaggerated sticking behavior to the blade (Rathod et al. 2025). The data from the present study indicated that the mozzarella remained cohesive but became increasingly adhesive to the shredding blade as storage progressed.

#### 3.3.3 Sensory Texture and TPA Correlation

While sensory texture attributes and TPA physical properties are distinct measures, relationships among the measures were assessed through PLS regression. On the loading plot (Figure S1), samples varying by treatment (control and modified) and storage time (5, 25, 50, and 75 days) were plotted with the sensory texture attributes and TPA physical properties. The model created in the present study was weak, as *Q* ^2^ was 0.152 and *R* ^2^ was 0.334. The *Q* ^2^ and *R* ^2^ values are important in understanding the goodness of fit of the model, with values lower than 0.5 suggesting low predictive power (Di Monaco et al. 2008; Wold et al. 2001). The low predictiveness of the PLS model suggests a weak relationship between physical sensory texture attributes and TPA physical properties, implying their distinct nature. This may be due to a lack of training in the panelists, leading to increased variation.

Supporting previous results, mozzarella samples were grouped by storage time. Day 5 samples (control and modified) were defined by their lower springiness, gumminess, chewiness, and hardness, all TPA determinations. These TPA determinations were in contrast with Day 50 and Day 75 mozzarella cheese, with higher chewdown adhesiveness and adhesiveness (determined by TPA). Day 25 samples were defined by their hand springiness and hand firmness.

Weak relationships between the TPA measurements and sensory panel assessments were made through this model. Negative correlations were observed between sensory texture attributes and physical properties, which are indicated by *R* ^2^ values less than −0.80 and *p* values less than 0.05 (Walsh et al. 2020). Sensory chewdown adhesiveness and cohesiveness were significantly negatively correlated with TPA hardness on Cycle 1 (*R* ^2^ = −0.84 and *R* ^2^ = −0.85, respectively) and springiness (*R* ^2^ = −0.84 and *R* ^2^ = −0.85, *p* ≤ 0.05, respectively). Sensory chewdown adhesiveness was also negatively correlated with gumminess (*R* ^2^ = −0.84, *p* ≤ 0.05) and chewiness (*R* ^2^ = −0.88, *p* ≤ 0.05). Gumminess and chewiness were negatively associated with sensory chewdown cohesiveness (*R* ^2^ = −0.76 and *R* ^2^ = −0.78, *p* ≤ 0.05, respectively).

Examples of these findings include that as TPA hardness increased in the mozzarella samples, sensory perception of adhesiveness and cohesiveness both decreased. This suggested that panelists perceived less sticking of the mozzarella samples to the surfaces of the mouth and less sticking together of the bolus itself, a behavior that was observed with Day 5 of storage for both samples. This behavior is consistent with harder mozzarella samples, which display an intact casein structure that breaks down with proteolysis during storage. In addition, springiness in the mozzarella was associated with a decreased sensory perception of cohesiveness and adhesiveness. These results provide evidence of the progression of proteolysis leading to a structure capable of less recovery when under applied stress (To et al. 2022). The present study also found that an increase in sensory chewdown adhesiveness was associated with decreased TPA gumminess and chewiness, a behavior seen previously in mozzarella literature (Foegeding and Drake 2007). A previous study reported a positive correlation, thus highlighting the complex relationship between instrumental measurements of sticky attributes and sensory analysis (Sandoval‐Copado et al. 2016; Półtorak et al. 2015).

Although sensory texture attributes and TPA physical properties can be correlated, there are challenges due to the measurement methodology. While TPA measures physical properties with the two‐bite compression test, it is not fully capable of predicting the entire sensory perception of texture (Foegeding and Drake 2007). In combination, sensory texture evaluation and TPA can provide a fuller picture of the properties and sensory perception of cheese and can be further supported through methods such as large amplitude oscillatory shear testing or small amplitude oscillatory shear (Zhu et al. 2015).

### 3.4 Melted Mozzarella Analyses

#### 3.4.1 Trained Panel Sensory Analysis

In evaluating 15 sensory attributes, the one significant attribute difference (*p* ≤ 0.05) detected between control and modified treatment of melted mozzarella was bitterness (Table S4). Bitterness, an undesirable attribute in mozzarella as an off taste, was significantly higher in the modified treatment at (4.41 on a 15‐cm line scale) compared to the control treatment (4.11 on a 15‐cm line scale), averaged over all storage times. This difference may not be detected by general consumers, however, and would need to be further examined in a consumer study. There was no interaction between storage time and treatment for bitterness. This increase in bitterness over storage time is attributed to the continued proteolysis (Moynihan et al. 2014; Banville et al. 2013).

Storage time influenced more sensory attributes than treatment, including the intensities of butter aroma, yogurt aroma, salt, acid, bitter, butter flavor, yogurt flavor, first‐chew hardness, chewdown cohesiveness, and chewdown chewiness (*p* ≤ 0.05) (Table 5). While these trends may not be linear, refresher training of panelists before each storage point may have reduced some variation between individual panelists. As storage time increased, butter and yogurt aromas increased, with differences noted between Day 5 and Day 50 of storage. Salt intensities decreased throughout storage, and acid intensities were highest at Day 25 compared to the other storage days (*p* ≤ 0.05). Bitter intensities were higher at Days 50 and 75 of storage compared to Day 5 of storage (*p* ≤ 0.05). Similar to butter aroma, butter flavor increased over storage between Day 5 and Day 50, and yogurt flavor increased between Day 5 and Day 25. For texture, first‐chew hardness was higher at Days 5 and 25 of storage compared to the later storage days of 50 and 75 (*p* ≤ 0.05). Chewdown cohesiveness initially increased between Day 5 and Day 25 but then decreased in intensity. Chewdown chewiness intensity values decreased over the entire storage period, with the lowest values at Day 75 of storage (*p* ≤ 0.05).

**TABLE 5:** Mean values of significant sensory attributes (identified using ANOVA) of melted mozzarella cheese stored for 5, 25, 50, and 75 days at 3.3°C. Samples were evaluated by a trained sensory evaluation panel (*n* = 10) using a 15‐cm line scale for attribute intensity. Data were collapsed across treatment, replicate batches, and panelists, and analyzed using Tukey's HSD. Different letters within a column represent a significant difference among cheese treatments for a given parameter (*p* ≤ 0.05).

	Aroma		Taste/flavor					Texture		
Storage day (3.3°C)	Butter aroma	Yogurt aroma	Salt	Acid	Bitter	Butter flavor	Yogurt flavor	First‐chew hardness	Chewdown cohesive	Chewdown chewiness
5	7.04 ± 1.94 a	6.92 ± 1.12 a	9.55 ± 1.57 a	7.84 ± 1.48 ab	3.75 ± 1.35 a	6.88 ± 1.80 a	7.29 ± 1.17 a	4.80 ± 1.49 a	7.83 ± 2.10 a	6.95 ± 1.92 a
25	7.55 ± 1.21 cb	7.52 ± 0.98 b	9.42 ± 1.75 ab	8.11 ± 1.14 b	4.015 ± 1.81 a	7.27 ± 1.08 ab	7.73 ± 0.96 b	4.20 ± 1.77 b	9.05 ± 1.41 b	6.09 ± 1.52 b
50	7.77 ± 0.73 c	7.53 ± 0.78 b	9.02 ± 1.53 ab	7.58 ± 1.05 a	4.64 ± 1.48 b	7.42 ± 0.95 b	7.72 ± 0.87 b	3.21 ± 1.48 c	8.38 ± 2.11 ab	5.79 ± 2.21 b
75	7.30 ± 1.16 ab	7.16 ± 0.75 a	8.87 ± 1.63 b	7.60 ± 1.28 a	4.60 ± 1.20 b	7.01 ± 1.40 ab	7.48 ± 1.03 ab	3.14 ± 1.30 c	7.69 ± 2.38 b	4.06 ± 1.75 c

**FIGURE 4:**
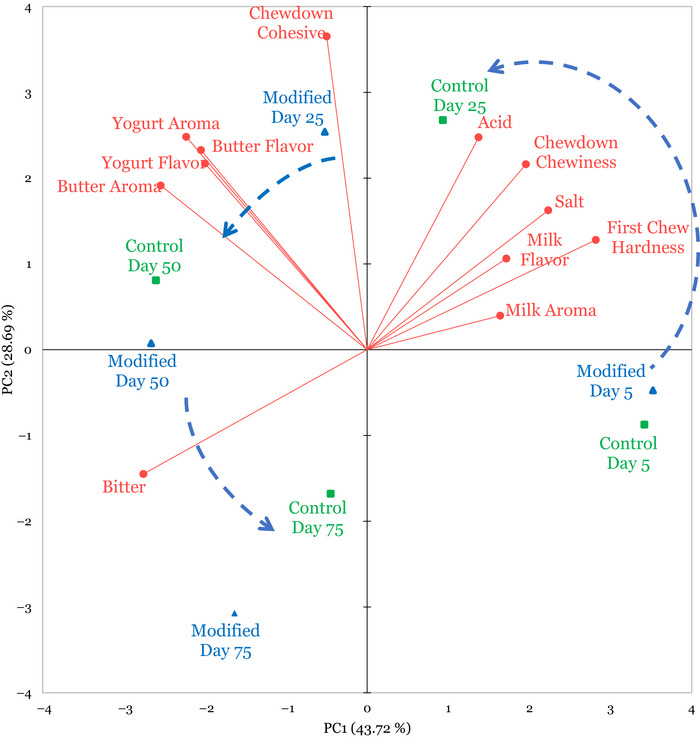
Principal component analysis of melted Mozzarella cheese samples stored for 5, 25, 50, and 75 days at 3.3°C, as assessed by a trained sensory panel (*n* = 10 panelists). Sensory attributes (red vectors) were assessed along a 15‐cm line scale, and results were collapsed by replicate batches. The control mozzarella samples without added adjunct culture are presented in blue, and the modified mozzarella samples with added adjunct culture are presented in green.

To visualize the relationship among sensory properties of melted mozzarella and sample treatment and storage time, a PCA plot was generated (Figure 4). This PCA explained 72.41% of the variation among the data. PC1 (describing 43.72% of the variation) was described by the contrast between milk aroma and bitterness, while PC2 (describing 28.69% of the variation) was primarily described by chewdown cohesiveness and acidity.

PCA showed a separation of samples over storage time. The treatments at Day 5 were located in the bottom right quadrant of the PCA plot and were defined by high milk aroma intensities and low bitterness. When the samples were evaluated on Day 25, both treatments shifted into the upper quadrants of the PCA plot and were associated with increased cohesiveness upon chewdown, as well as decreased milk aroma/flavor and a lower intensity of bitterness. The modified mozzarella cheese sample from Day 25 was also associated with higher butter flavor and yogurt aroma/flavor. When progressing to Day 50 of storage, both treatments moved closer to the *x*‐axis, with the control and modified treatments in the upper left quadrant, characterized by their increased bitterness and butter aroma, along with decreased milk aroma and acidity. Finally, both Day 75 treatments migrated to the bottom left quadrant and were defined by their high bitterness and low cohesiveness with mastication.

In the melted mozzarella, the progression of storage time led to changes in nearly all the attributes measured by the trained panel. Each storage day was distinctly defined by a set of characteristics, indicative of the aging process taking place over the 75 days of storage. In early storage, both treatments were mostly defined by their milk aroma, milk flavor, and first‐chew hardness. This milk aroma is characteristic of high‐moisture young mozzarella, as the short aging period leaves less time for more complex flavor development seen in cheeses like Cheddar or Gouda (Yang et al. 2025; Natrella et al. 2020). As storage progressed, the melted cheese samples increased in their acidity, butter attributes, and yogurt attributes. The increase in acidity can be attributed to the continued activity of the starter culture and adjunct culture, fermenting lactose into lactic acid. After lactose is fully removed via fermentation, additional ketones produced can contribute to the yogurt flavor (Yang et al. 2025). The increase in these attributes is indicative of continued fermentation through pathways producing diacetyl, acetoin, lactic acid, and acetic acid, compounds responsible for these aromas and flavors (Li et al. 2024). By the end of storage, the sensory evaluation of melted samples noted decreases in the texture attributes of hardness and adhesiveness. The change in these attributes was attributed to the continued proteolysis that takes place over storage (Banville et al. 2014; Moynihan et al. 2014).

#### 3.4.2 Meltability

**FIGURE 5:**
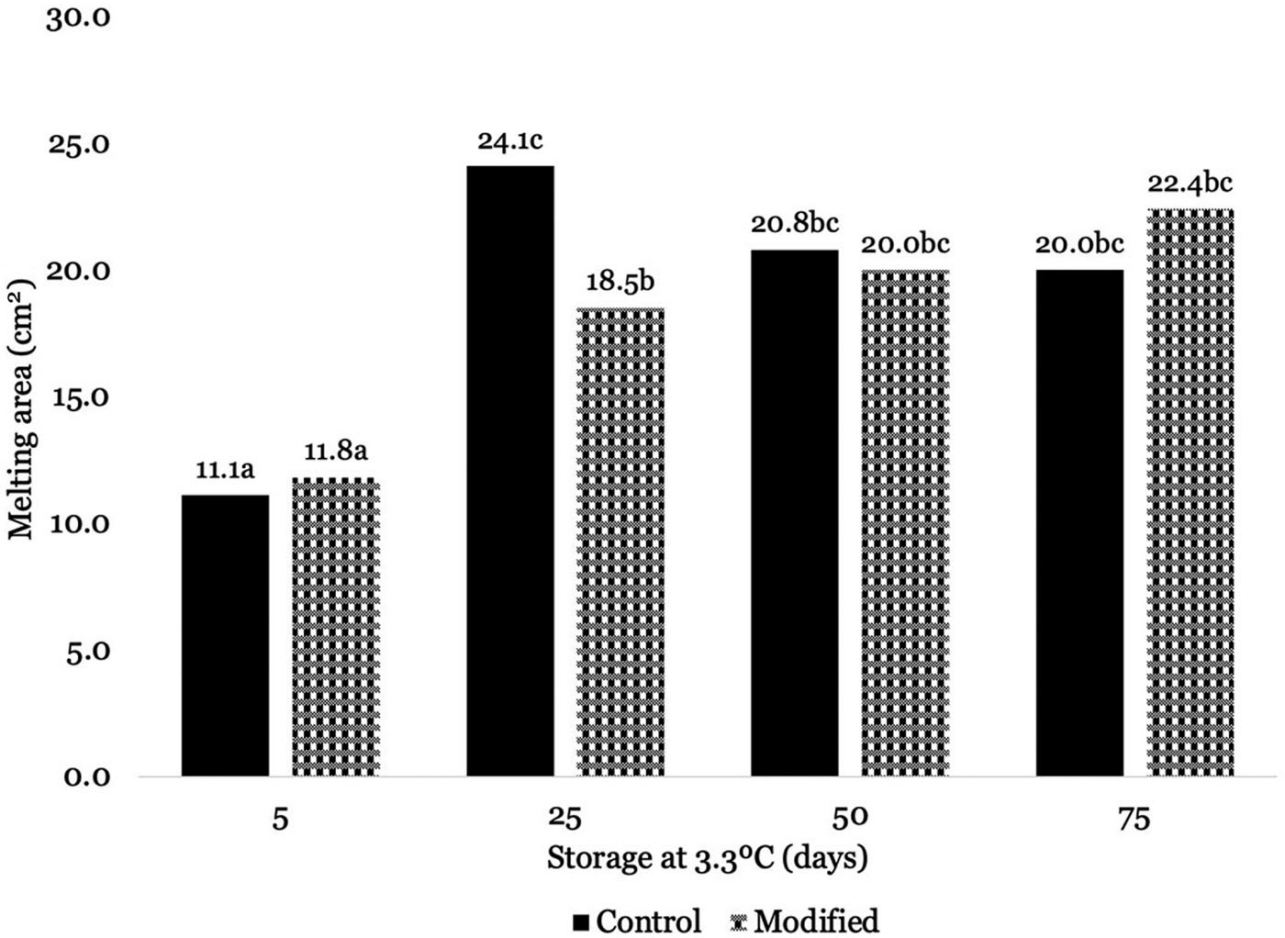
Mean values of melted area (cm^2^) of mozzarella cheese samples stored for 5, 25, 50, and 75 days at 3.3°C, as evaluated by a modified Schreiber test and analyzed using Tukey's HSD. Data were collapsed across replicate batches. Different letters within a column represent a significant difference. Control mozzarella samples did not contain adjunct culture, and modified mozzarella samples did contain adjunct culture.

The melting properties of the shredded mozzarella cheese samples were also assessed instrumentally by comparing total area before and after heating (cm^2^). There was a significant interaction between treatment and storage time of the final melted area of the cheese treatments (Figure 5). On Day 5 of storage, both mozzarella treatments had significantly lower melted areas compared to all other storage times. On Day 25, the melted area of the control mozzarella increased significantly, resulting in a final melted area that was double that of Day 5. Similarly, the melted area of the Day 25 modified mozzarella was significantly higher than that of Day 5, though only increasing by half. This presented as a significant difference between the two treatments in melting behavior on Day 25 of storage. Following Day 25 of storage, the melting of the control treatments decreased slightly, whereas the modified treatment increased; however, these changes were not significant (*p* ≥ 0.05).

Meltability increased over storage time, with the addition of the *Lacti. casei* adjunct culture increasing melting to a lesser degree than the control mozzarella on Day 25 of storage. During early storage (Day 5), both the control and modified mozzarella exhibited melting behavior characteristic of young mozzarella, with little change due to heat application (Kindstedt 1993). Due to the part‐skim FDM content of the produced mozzarella being below 45% (w/w), the meltability in early storage may have been lower than that of typical LMPS mozzarella, as meltability has been found to increase with higher FDM and lower protein, total, and insoluble calcium contents (Lefevere et al. 2000; Tunick et al. 1991, 1993). The meltability of both mozzarella treatments increased at Day 25 of storage, with differences observed between the control and adjunct treatments. This increase in meltability in the adjunct culture mozzarella may be attributed to the additional proteolysis by *Lacti. casei* together with the proteolysis contributed by *S. thermophilus*, allowing for the redistribution and equilibration of components such as calcium, protein, and serum in the casein matrix. This redistribution allows the fat globules present in the cheese to evenly heat and allows the matrix to melt (Gonçalves and Cardarelli 2020). The slight reduction in the modified mozzarella melting with the addition of *Lacti. casei* adjunct culture at Day 25 of storage may mean that the modified mozzarella may be stored longer before reaching the point of overmelting. Overmelting leads to soupy or flow‐off‐the‐crust behavior when melted, both of which negatively impact the pizza or baked applications of mozzarella (Rizzi et al. 2023). However, this reduction is slight and may not be noticeable to end users even when detectable in a commercial context. For example, if the mozzarella is applied in lasagna, overmelting may be less detectable, but in pizza applications, the overmelting can be a visual negative attribute for consumers.

## 4 Limitations

This study demonstrated that the application of *Lacti. casei* adjunct culture during mozzarella cheesemaking did not profoundly affect sensory or physical attributes but did modify the melting properties of mozzarella cheese. However, several limitations must be noted.

The present study evaluated part‐skim, pasta filata mozzarella. Future studies may explore different compositions and production methods of mozzarella, and the influence that an adjunct culture has on these parameters, as part‐skim, pasta filata is limited in production compared to the more popular LMPS mozzarella. In the present study, the mozzarella was produced at pilot‐scale batches. Due to the pilot scale, the milk was not standardized for protein or protein‐to‐fat ratio prior to cheesemaking. In order to account for this limitation, the final fat, protein, and moisture were measured for all produced batches. There are potential concerns with the addition of adjunct culture, specifically gas formation and the gassy defect found with some *Lactobacillus* cultures (O'Sullivan et al. 2016). These should be examined in mozzarella in future adjunct culture experiments, as they can be major detractors for producers. Additionally, culture isolation utilizing additional media or identification methods could have better isolated *Lacti. casei* when compared with MRS‐V agar, and should be considered in future experiments.

Different results may be observed with scale‐up, as the provided cultures were specifically designed for industry‐size batches rather than the pilot‐scale batches produced. Another limitation regarding processing was the lack of a stretching machine. The use of screw stretchers or extruders has become near universal in large‐scale mozzarella production. While the hand stretching procedure was standardized across the four individuals, the process may still have introduced additional variation. One approach to better understand the impact of these stretching methods would be to directly compare the traditional and industry methods. This could provide key information about differences in industrial mozzarella processing, a field that is already being explored with variation in stretching temperature (Gonçalves and Cardarelli 2020; Natrella et al. 2022b). Additionally, comparisons between pasta filata and stirred curd techniques with considerations of adjunct culture could provide improvements for pizza cheese.

As *Lacti. casei* shows promise in the baked applications, future work should be focused on the application in a mozzarella model with a descriptive panel. A panel of at least eight individuals would be considered robust for discrimination between samples, along with proper training (Heymann et al. 2012). Baked pizza attributes could be assessed, including blister color, blister coverage, oiling off, moisture release, and stretch behaviors. A consumer panel could be beneficial in future work to better understand the consumer desires for pizza cheese attributes. Comparisons between mozzarella of varying fat contents (reduced fat, part skim, and full fat) with the inclusion of adjunct culture could address difficulties with baking behavior.

Future research could also include other chemical and physical measurements. Volatile compound profiling could be accomplished through gas chromatography–mass spectrometry to quantify changes in the aroma profile, such as changes in buttery compounds like diacetyl and acetoin (Natrella et al. 2020, 2022b). The extent of proteolysis in the mozzarella cheese could also be collected using methods that measure water‐soluble nitrogen (Ozturk et al. 2018). Methods such as small‐amplitude oscillatory shear (SAOS) or large‐amplitude oscillatory shear (LAOS) could also be used to better characterize the meltability of the mozzarella through understanding and quantifying the viscoelastic behavior of the mozzarella (Zhu et al. 2015).

## 5 Conclusions

The current study assessed the impact of a *Lacticaseibacillus casei* adjunct culture in part‐skim, pasta filata mozzarella cheese, focusing on physical, sensory, and functional attributes over refrigerated storage. Overall, the addition of *Lacti. casei* did not negatively or positively impact the physical properties or sensory attributes of cold mozzarella cheese, as the part‐skim mozzarella produced behaved as expected over the 75‐day storage period. Meltability was influenced by the presence of the adjunct culture and storage time, with both control and modified cheeses increasing in meltability over storage.

The addition of *Lacti. casei* as an adjunct culture in part‐skim mozzarella did not detract from sensory or functional quality and may offer benefits in baked applications by modulating melting behavior over storage time. These findings support the potential use of *Lacti. casei* in mozzarella formulations where improved or consistent melting performance is desired, without compromising consumer expectations for flavor, texture, or appearance. Cheese producers may opt for *Lacti. casei* addition as a cost‐saving measure. Without the additional cost of cultures, producers will not have to invest in further pilot‐scale or large‐scale testing and therefore maintain current production costs.

## Author Contributions

**Katherine Rehberger**: investigation, writing – original draft, methodology, data curation, formal analysis, writing – review and editing, validation, visualization. **Stephanie Clark**: conceptualization, methodology, writing – original draft, writing – review and editing, visualization, investigation. **Gülhan Ünlü**: conceptualization, methodology, formal analysis, visualization, writing – original draft, investigation. **Helen Joyner**: conceptualization, methodology, visualization, writing – original draft, investigation. **Carolyn F. Ross**: conceptualization, methodology, data curation, investigation, validation, formal analysis, supervision, resources, project administration, funding acquisition, writing – original draft, visualization.

## Funding

Funding was received from BUILD Dairy and Pacific Coast Coalition—Dairy Business Innovation Initiative. This research was also supported in part by IFF through the donation of all cheese cultures. The authors also acknowledge the support of the USDA National Institute of Food and Agriculture Hatch project under accession #101636.

## Ethics Statement

The study was approved by the Washington State University Institutional Review Board for human subject participation under IRB# 20397‐001, with written informed consent obtained from all study participants. All aspects of data collection and storage complied with the standards specified by this body.

## Conflicts of Interest

The authors declare no conflicts of interest.

## Supporting information




**Supplementary Materials**: jfds71095‐sup‐0001‐SuppMat.docx

## Data Availability

The data that support the findings of this study are available from the corresponding author upon reasonable request.
