# Supplementary material for: Sensory, Physical, and Functional Properties of Part‐Skim, Pasta Filata Mozzarella Made With or Without Lacticaseibacillus casei Adjunct Culture
Source: J Food Sci. 2026 May 8;91:e71095. doi: 10.1111/1750-3841.71095 (PMC13155180; doi:10.1111/1750-3841.71095)
Supplement: Supplementary file 1 — Supplementary Materials: jfds71095‐sup‐0001‐SuppMat.docx [file JFDS-91-0-s001.docx]

SUPPLEMENTARY TABLES AND FIGURES

**Table S1.** Two-way ANOVA comparison of milk fat% by treatment and production day. Data were analyzed using a two-way ANOVA between treatment and production day. An * represents a significant difference (p<0.05) in a given attribute.

| **Source of Variation** | **SS** | **df** | **MS** | **F** | **p-value** |
| --- | --- | --- | --- | --- | --- |
| **Treatment** | 0.01815 | 1 | 0.01815 | 9.81 | 0.089 |
| **Batch Day** | 0.0589 | 2 | 0.02945 | 15.92 | 0.059 |
| **Error** | 0.0037 | 2 | 0.00185 |  |  |
| **Total** | 0.08075 | 5 |  |  |  |

**Table S2.** Influence expressed as F-values of storage time (5, 25, 50, 75 days at 3.3°C), treatment (presence or absence of adjunct culture in cheese), panelist (n=10), and storage time*treatment on trained sensory panel assessment of cubed mozzarella cheese with and without adjunct culture treatment. Data were analyzed using a three-way ANOVA between storage time, treatment, and panelist. An * represents a significant difference (p<0.05) in a given attribute.

| **Source of Variation** | **Storage Time** | **Treatment** | **Panelist** | **Storage Time x Treatment** |
| --- | --- | --- | --- | --- |
| Milk Aroma | 2.26 | 0.18 | 28.29* | 0.81 |
| Butter Aroma | 3.95* | 0.05 | 25.64* | 0.24 |
| Yogurt Aroma | 1.77 | 2.37 | 37.39* | 1.13 |
| White Color | 16.83* | 0.37 | 9.26* | 0.48 |
| Yellow Color | 8.48* | 2.02 | 8.80* | 0.42 |
| Surface Uniformity | 3.00* | 1.73 | 41.87* | 0.51 |
| Salt | 2.30 | 1.01 | 19.18* | 1.05 |
| Acid | 1.62 | 0.30 | 29.54* | 0.65 |
| Bitter | 14.91* | 0.43 | 32.28* | 0.29 |
| Milk Flavor | 2.27 | 0.03 | 28.44* | 2.62 |
| Butter Flavor | 20.91* | 0.70 | 43.27* | 0.25 |
| Yogurt Flavor | 2.84* | 0.31 | 37.69* | 0.54 |
| Hand Firmness | 6.44* | 0.90 | 8.15* | 0.24 |
| Hand Springiness | 2.50 | 3.85 | 22.52* | 1.23 |
| First Chew Hardness | 2.31 | 1.55 | 10.15* | 0.37 |
| Chewdown Adhesive | 20.47* | 0.16 | 36.84* | 0.51 |
| Chewdown Cohesive | 16.18* | 0.48 | 27.98* | 1.18 |
| Chewdown Chewiness | 0.80 | 0.25 | 16.07* | 0.30 |

**Table S3.** Mean values of Texture Profile Analysis attributes of mozzarella cheese with and without adjunct culture treatment. Data were collapsed across treatment, storage time, and replicate batches, and analyzed using Tukey’s HSD. Different letters within a column represent a significant difference among mozzarella cheese samples by storage day for a given parameter (p≤0.05).

| **Treatment** | **Hardness cycle 1 (g)** | **Hardness cycle 2 (g)** | **Springiness (mm)** | **Gumminess (g)** | **Chewiness (mJ)** | **Adhesiveness (mJ)** | **Cohesiveness** |
| --- | --- | --- | --- | --- | --- | --- | --- |
| Control | 2818a | 1979a | 4.94a | 946.1a | 50.04a | 2.38a | 0.33a |
| Modified | 2596a | 1798a | 5.02a | 878.8a | 44.88a | 2.41a | 0.34a |

**Table S4.** Influence expressed as F-values of storage time (5, 25, 50, 75 days at 3.3°C), treatment (presence or absence of adjunct culture in cheese), panelist, and storage time*treatment on trained sensory analysis panel (n=10) assessment of melted mozzarella cheese with and without adjunct culture treatment. Melted cheese was prepared by heating mozzarella cheese (20 g) in microwave for 15 seconds. Data were analyzed using a three-way ANOVA between storage time, treatment, and panelist. An * represents a significant difference (p<0.05) in a given attribute.

| **Source of Variation** | **Storage Time** | **Treatment** | **Panelist** | **Storage Time x Treatment** |
| --- | --- | --- | --- | --- |
| Milk Aroma | 0.19 | 0.34 | 22.94* | 0.81 |
| Butter Aroma | 7.44* | 0.28 | 27.09* | 1.04 |
| Yogurt Aroma | 9.51* | 1.49 | 29.57* | 0.72 |
| Salt | 4.42* | 0.01 | 24.63* | 0.82 |
| Acid | 3.50* | 0.34 | 20.82* | 0.24 |
| Bitter | 5.66* | 4.16* | 18.82* | 1.59 |
| Milk Flavor | 0.51 | 1.61 | 42.29* | 2.22 |
| Butter Flavor | 4.36* | 1.04 | 31.08* | 0.55 |
| Yogurt Flavor | 3.99* | 0.02 | 21.52* | 1.09 |
| First Chew Hardness | 42.01* | 0.96 | 35.55* | 0.86 |
| Chewdown Adhesive | 3.38* | 1.42 | 20.83* | 1.44 |
| Chewdown Chewiness | 24.96* | 0.47 | 14.90* | 1.82 |

**Figure S1.** Partial Least Squares (PLS) regression biplot correlating TPA physical properties (red dots) evaluated by trained panel (n = 10) to sensory texture attributes (gold dots) for mozzarella samples (blue dots = control (starter culture only) samples, green dots = modified (starter + adjunct culture) samples) described by their storage time (5, 25, 50, and 75 days) at 3.3ºC.
